# Psychosocial interventions for adolescents in forced migration: A scoping review protocol

**DOI:** 10.1371/journal.pone.0349506

**Published:** 2026-05-19

**Authors:** Damilola Onietan, Bala Isa Harri, Temitayo Sodunke, Omolayo Anjorin, Tegwende Seedu, Andem Effiong Etim Duke, Ejemai Eboreime

**Affiliations:** 1 Department of Psychiatry, Dalhousie University, Halifax, Nova Scotia, Canada; 2 School of Health and Human Performance, Dalhousie University, Halifax, Nova Scotia, Canada; 3 Dalhousie University Faculty of Medicine, Halifax, Nova Scotia, Canada; Yunnan University, CHINA

## Abstract

**Background:**

Adolescents account for 40% of forcibly displaced people worldwide and are often the most vulnerable to mental health challenges. Although psychosocial interventions are becoming popular, there remains a significant gap in the application of psychosocial interventions for adolescents in forced migration context. Mapping the existing literature is necessary to help guide the use of psychosocial interventions for this population.

**Objective:**

To systematically map and synthesise the scope of existing literature related to psychosocial interventions for adolescents in forced migration situations. This review will inform the policy and practice.

**Inclusion criteria:**

This review will include empirical studies using qualitative, quantitative, or mixed methods approaches. The review will include any study where adolescents aged 10–19 years in forced migration settings receive psychosocial intervention with the purpose of improving psychological well-being, mental health, or psychosocial functioning. The study population will include adolescent refugees, asylum seekers, internally displaced persons, and other adolescents who have been compelled to leave their homes due to conflict, persecution, natural disasters, or other threats to safety and security. Studies will include both high-income and low- and middle-income countries. Only studies published from 2000 to the present will be included in the review.

**Method:**

The review will follow the Joanna Briggs Institute methodology and PRISMA-ScR reporting guidelines. It will include a comprehensive search of published studies in databases and grey literature. Two independent reviewers will evaluate all abstracts and full articles that are written in English. We will identify concepts from psychosocial interventions, categorise accepted studies according to characteristics, describe implementation factors, and report outcomes of selected studies.

**Scoping review registration:**

This study protocol is registered with Open Science Framework (OSF) with DOI 10.17605/OSF.IO/ADF92.

## Introduction

Forced migration is a pervasive global phenomenon that has increased over recent decades due to escalating geopolitical conflicts, environmental disasters, systemic violence, and persecution. It is a traumatising event related to separation and loss, loneliness, a lack of a sense of belonging, threats towards one’s identity, developmental regression and grief [1]. Currently, there are 123.2 million forcibly displaced people, including refugees, asylum-seekers, other people in need of international protection and internally displaced people [2]. Among the staggering number of forcibly displaced people worldwide, children and adolescents below 18 years account for 49 million [2]. These individuals are facing immense challenges throughout their journeys and in host communities.

Adolescents (typically defined as individuals aged (10–19 years) represent a particularly vulnerable demographic in forced migration [3]. Adolescence is a transitional life phase inherently characterised by identity formation, increased autonomy, emotional regulation development, and peer affiliation, all of which are susceptible to disruption in the context of forced migration [4]. Adolescents affected by forced migration may include refugees, asylum seekers, stateless persons, internally displaced persons (IDPs), and individuals in protracted displacement situations. Adolescents in a forced migration setting often face cumulative and intersecting adversities, including but not limited to Sexual and Gender-Based Violence (SGBV), physical violence, psychological and emotional violence, trafficking and exploitation, violence during transit, trauma, bereavement, separation from family or caregivers, prolonged uncertainty regarding legal status, educational disruption, marginalisation, and acculturation stress [5,6].

Protracted conflicts, such as the ongoing crises in Sudan and Somalia, and the wars in Ukraine and the genocide in Gaza, have significantly exacerbated the challenges faced by forcibly displaced adolescents [7,8]. Studies have shown that there is a higher prevalence of mental health conditions among this population compared to their non-displaced peers and even adult refugees [9]. Unlike short-term crises, prolonged or intergenerational exposure to violence, instability, and displacement leads to cumulative and chronic stress, profoundly impacting mental health and well-being [9].

The prolonged uncertainty, lack of safety, legal and economic barriers and limited access to basic services such as health services in protracted displacement settings contribute to a heightened risk of developing complex mental health conditions. The common mental health conditions include post-traumatic stress disorder (PTSD), depression, anxiety, and other stress-related disorders [10–12]. These long-term stressors can manifest as difficulties in emotional regulation, social withdrawal, academic struggles, behavioural problems, and, in severe cases, suicidal ideation [13,14]. Factors contributing to these outcomes include pre-migration trauma (e.g., exposure to violence, torture), peri-migration stressors (e.g., dangerous journeys, separation from family, exploitation), and post-migration challenges (e.g., discrimination, acculturation stress, poverty, lack of access to education and healthcare, uncertain legal status) [15]. Therefore, the need for psychosocial interventions is thus amplified in these contexts, requiring sustained and comprehensive support tailored to the unique and evolving needs of adolescents living in prolonged states of forced migration [16].

Psychosocial interventions in this context are operationally defined as structured, purposeful activities, programmes, or therapeutic approaches that have an explicit primary objective of improving psychological well-being, mental health, and/or psychosocial functioning among target populations. This definition draws on the Inter-Agency Standing Committee (IASC) framework for Mental Health and Psychosocial Support (MHPSS), which conceptualises interventions along a tiered pyramid [17]. They may include, but are not limited to, Cognitive-Behavioural Therapy (CBT), Trauma-Focused Cognitive-Behavioural Therapy (TF-CBT), Narrative Exposure Therapy (NET), and other manualised psychotherapeutic approaches [18,19]. The second category includes school-based mental health programmes, structured peer support groups, family-based psychosocial interventions, group psychosocial support programmes, and community-based [20–22]. Despite the evident need, access to appropriate and effective psychosocial interventions for adolescents in forced migration remains a significant challenge.

Global frameworks such as the IASC Guidelines on Mental Health and Psychosocial Support in Emergency Settings emphasise the need for layered and context-sensitive care, the operationalisation of these guidelines for adolescents remains inconsistent [17]. Barriers include a lack of age-specific models, insufficient community engagement, fragmented service delivery, language differences, cultural insensitivity of services, stigma associated with mental health issues, a lack of trained professionals, and systemic limitations in host countries [17]. Mapping this evidence base will not only identify existing knowledge gaps but also inform the development of age-appropriate, culturally responsive, and contextually grounded psychosocial interventions.

Some systematic reviews have synthesised evidence on the impact of psychosocial interventions for this population, but there is a need for a scoping review that systematically maps the breadth of available evidence, identifies key characteristics of interventions, and highlights research gaps. A 2020 systematic review included seven studies focused on psychosocial interventions for newly arrived adolescent refugees [23]. The review found that psychosocial interventions such as music education programs, a standardised anxiety prevention program, had positive effects on the psychological symptoms of anxiety, post-traumatic stress disorder, and depression, as well as on the personal development of newly arrived adolescents regarding their life satisfaction. While valuable, this review’s scope was limited to the initial arrival period, and it did not aim to comprehensively map all types of psychosocial interventions across different phases of forced migration. School-based programmes and cognitive-behavioural therapies have demonstrated effectiveness in promoting mental health and well-being among adolescents [24,25].

Another relevant scoping review aimed to map existing research into school-based mental health interventions for migrant children and young people [21]. While valuable for understanding school-based approaches and forced migration, this scoping review focuses specifically on interventions within a school setting. This highlights a potential gap that the proposed scoping review can fill by examining a wider range of psychosocial interventions beyond the school environment, and with a specific focus on adolescents in forced migration

Generally, adolescent-specific psychosocial interventions remain comparatively understudied within the literature. Most existing research tends to aggregate data across age groups or focuses primarily on either children or adults, thereby obscuring the developmental specificity and distinct psychosocial needs of adolescents [12].

A Scoping review is particularly suitable for emerging fields of inquiry, where the research questions are broad and heterogeneous study designs are anticipated [26,27]. First, the research questions in this review are broad, aiming to map the types, characteristics, theoretical frameworks, delivery formats, and implementation contexts of psychosocial interventions for adolescents in forced migration, rather than evaluating the effectiveness of a specific intervention. Second, the evidence base in this field is methodologically heterogeneous, spanning clinical trials, qualitative studies, programme evaluations, pilot studies, and grey literature, a diversity that scoping reviews are designed to accommodate without requiring comparable effect sizes or standardised outcome measures [26]. Third, a key objective is to identify gaps in the literature on psychosocial interventions for adolescents in forced migration contexts; scoping reviews are specifically designed for this purpose and to inform future systematic reviews [26,27]. Lastly, the interest in mapping implementation factors, facilitators, barriers, and delivery contexts across diverse humanitarian and resettlement settings requires a methodology capable of synthesising findings across fundamentally different contexts [27]. Accordingly, this review will systematically map the breadth of existing evidence on psychosocial interventions for adolescents in forced migration. In turn, this review can support practitioners, policymakers, and researchers working to promote resilience, recovery, and long-term well-being in one of the most vulnerable yet often overlooked populations in humanitarian and post conflict settings.

### Objectives

A preliminary search of PROSPERO, CINAHL, and the Cochrane Database of Systematic Reviews was conducted, and no current or underway scoping reviews on the topic were identified. Therefore, this scoping review aims to map and synthesize existing evidence on psychosocial interventions designed for or implemented with adolescents in forced migration situations globally. This review will examine the geographic distribution of included studies to assess the global scope of evidence on forced migration. Regional gaps and underrepresented areas within the evidence base will be identified and mapped. Specifically, we will identify the types, characteristics, and theoretical frameworks underlying these interventions; analyze their settings, delivery methods, and implementation contexts; examine reported outcomes and effectiveness measures; and identify gaps in current research and practice, including facilitators and barriers to implementation.

### Review question

**Primary question:** What psychosocial interventions have been developed, implemented, or evaluated for adolescents in forced migration contexts?

**Secondary research questions:**

1. What outcomes have been measured and reported in utilising psychosocial interventions for adolescents in forced migration settings?
2. What are the reported facilitators and barriers to implementing psychosocial interventions for adolescents in forced migration settings?
3. What theoretical frameworks underpin the psychosocial interventions developed for adolescents in forced migration situations, and what delivery formats and settings have been utilised?
4. What populations of adolescents in forced migration have been included in research, and what groups remain understudied?

## Methods

This scoping review will be conducted according to the Joanna Briggs Institute (JBI) methodology for scoping reviews [27]. The review will follow the Preferred Reporting Items for Systematic Reviews and Meta-Analyses Extension for Scoping Reviews (PRISMA-ScR) guidelines [28].

### Eligibility criteria

We will include studies based on the Participants–Concept–Context (PCC) mnemonic recommended by the Joanna Briggs Institute (JBI) for scoping reviews [27].

#### Participants.

Studies must include adolescents aged 10–19 years who have experienced forced migration [29]. This population encompasses refugees, asylum seekers, internally displaced persons, stateless persons and other individuals who have been compelled to leave their homes due to conflict, persecution, natural disasters, or other threats to safety and security. Studies will be categorised into three groups based on how adolescent data are reported. The first group comprises studies in which all participants fall within the 10–19 age range. These will be included without restriction. The second group comprises mixed-age studies with disaggregated data, which are studies that include participants outside the 10–19 age range but report adolescent-specific data separately (e.g., separate subgroup analyses, stratified results, or independently reported outcomes for the adolescent subsample). These studies will be included, and only the adolescent-specific data will be extracted and synthesised. The third group comprises mixed-age studies without disaggregated data, which are studies that combine adolescent participants with children or adults without reporting age-disaggregated results. These studies will be included only if: (a) at least 75% of the study sample falls within the 10–19 age range, as determined by the reported mean or median age and age range of participants; or (b) the reported age range of the sample falls predominantly within the adolescent range (e.g., 12–21 years), such that the majority of participants can reasonably be assumed to be adolescents. Where neither condition is met, the study will be excluded. In cases where the age composition of the sample is unclear or insufficiently reported, the corresponding author of the study will be contacted to obtain clarification on the proportion of adolescent participants and, where possible, to request disaggregated data. All decisions regarding the inclusion of mixed-age studies will be documented and reported transparently, including the rationale for inclusion or exclusion in each case.

#### Concept.

Studies must examine a psychosocial intervention as defined in the operational criteria above. Specifically, to be eligible for inclusion, interventions must meet the following criteria: (i) the intervention must have an explicitly stated objective of improving psychological well-being, mental health, or psychosocial functioning; (ii) the intervention must target at least one recognised mental health or psychosocial outcome, such as reduction in symptomatology related to PTSD, depression, anxiety, or complicated grief; improvement in psychosocial functioning, emotional regulation, or social connectedness; or enhancement of psychological resilience and coping capacities [22,30]. Eligible interventions include, but are not limited to: clinical therapeutic interventions such as Cognitive-Behavioural Therapy (CBT), Trauma-Focused CBT (TF-CBT); structured psychosocial support programmes such as school-based mental health interventions, group psychosocial support, structured peer support, family-based psychosocial interventions, psychoeducation programmes, and creative or expressive arts-based interventions with explicit psychosocial objectives; and focused non-specialised supports such as Problem Management Plus (PM+), Life skills interventions, and psychosocial support delivered by trained non-specialists or community health workers.

#### Context.

The context includes any setting where adolescents in forced migration receive psychosocial interventions. This may consist of refugee camps, resettlement communities, urban settings, schools, healthcare facilities, community centres, or other formal and informal environments. Both high-income and low- and middle-income countries will be included, reflecting the global nature of forced migration.

#### Types of studies.

Both quantitative and qualitative study designs will be included, encompassing randomised controlled trials, quasi-experimental studies, cohort studies, case series, qualitative studies, and mixed-methods research. Program evaluations, implementation studies, pilot studies, and feasibility studies will potentially be included. The review will consider peer-reviewed journal articles, grey literature (including reports, theses, and conference proceedings) and policy documents. Only studies published in English will be included.

#### Exclusion criteria.

We will exclude studies that focus solely on adults (19 + years) or children (<10 years) without providing adolescent-specific data. We will also exclude studies addressing economic migrants or voluntary migration unless they specifically examine forced displacement. Studies examining purely physical health interventions that lack psychosocial components will be excluded, as will opinion pieces, editorials, or commentary without empirical data. Additionally, we will exclude studies that provide insufficient detail about intervention characteristics and duplicate publications of the same study. Interventions that investigate exclusively medical or pharmacological interventions will be excluded. Interventions that are primarily humanitarian, focusing on the provision of basic needs or protection-oriented, without incorporating psychosocial components, will be excluded [31]. This ensures the review captures interventions where psychosocial improvement is the intended purpose rather than an incidental outcome of broader programming.

#### Time period.

We will limit our search to studies published from January 2000 onwards, to capture contemporary approaches to psychosocial interventions developed in the post-millennium era. This timeframe aligns with pivotal developments in humanitarian mental health programming, such as the IASC Guidelines on Mental Health and Psychosocial Support in Emergency Settings and the emergence of scalable, evidence-based interventions specifically designed for conflict-affected and displaced populations [17,31]. This timeframe ensures we capture interventions developed using modern research methodologies and evidence-based practices while maintaining a manageable scope for comprehensive review. Studies published before 2000 may reflect outdated theoretical frameworks and intervention approaches that are less relevant to contemporary practice with forcibly displaced adolescents.

### Search strategy

A comprehensive three-step JBI-recommended search strategy will be employed [27]. The first step involves a preliminary search of two major databases. Specifically, MEDLINE and Scopus will be searched to identify relevant keywords, index terms, and controlled vocabulary. A comprehensive search strategy will be developed. Electronic databases to be searched include MEDLINE, PsycINFO (Ovid), CINAHL, Embase, Scopus (Elsevier) and ProQuest. We will also include grey literature from selected sources. (See Table 1). These databases have been selected for their collective capacity to yield broad evidence pertinent to the research topic, encompassing various interdisciplinary domains.

**Table 1:** List of search sources, including databases, conference proceedings, and grey literature.

Literature type	Sources
**Databases**	MEDLINE PsycINFO (Ovid) CINAHL Embase Scopus (Elsevier) ProQuest
**Grey literature**	Google scholar Open Grey UNHCR Global Digital Library WHO

The search terms will be refined and expanded to maximise the retrieval of relevant articles. Search strategies will incorporate Medical Subject Headings (MeSH) and context-specific terms related to three core concepts: adolescents, forced migration, and psychosocial interventions terms for adolescents will include “adolescent,” “teenager,” “youth,” “young people,” and age-specific terms. Forced migration terms will encompass “refugee,” “asylum seeker,” “internally displaced,” “forced migration,” “displacement,” and related concepts. Psychosocial intervention terms will include “psychosocial intervention,” “mental health intervention,” “therapy,” “counseling,” “psychological support,” and specific intervention types.

The tentative search strategy for Medline is as follows:

1. (adolescent/ or exp pediatrics/ or child, abandoned/ or exp child, exceptional/ or child, orphaned/ or child, unwanted/ or minors/ or (pediatric* or paediatric* or schoolchild* or boy or boys or girl* or middle school* or pubescen* or juvenile* or teen* or youth* or high school* or adolesc* or pre-pubesc* or prepubesc*).ti,ab,kf. or (child* or adolesc* or pediat* or paediat*).jn.
2. ((conflict* or forc* or war or involuntar* or violen*) adj3 (displace* or migrat* or migrant* or immigra* or refugee* or resettle*)).ti,ab,kf.
3. (idp or (refugee* adj3 (camp* or communit* or settle*)) or (asylum adj2 seek*) or (displaced adj2 (person* or people* or communit*))).ti,ab,kf.
4. #2 OR #3
5. #1 AND #4

In addition to database searches, proceedings from relevant grey literature sources will be examined to ensure a thorough exploration of available evidence [32]. (see Table 1). Reference lists of included studies will be manually searched to identify additional sources. Key authors in the field will be contacted to identify recent or unpublished work. Grey literature sources will be searched using terms derived from the three core concepts. Google Scholar searches will be limited to the first 200 results per string, sorted by relevance, consistent with established recommendations [32]. Organisational repositories, including the UNHCR publications database and the WHO institutional repository (IRIS), will be searched using internal search functions with terms related to adolescents, forced migration, and psychosocial interventions, adapted to each platform. OpenGrey will be searched for dissertations and theses using comparable terms. The same eligibility criteria applied to peer-reviewed literature will apply to grey literature screening. All search dates, terms, and records retrieved will be documented in a supplementary table to ensure transparency and reproducibility. The search will be limited to English-language publications to avoid potential discrepancies that occur during translation, as the research team is only fluent in English. The search in all databases will consist of studies from 2000 to the present. To maintain the currency of the review, searches will be updated to capture any new citations that emerge between the initial search and the final manuscript submission.

Authors of included studies will be contacted to obtain any missing or additional information necessary for clarification. Reference lists of all accepted studies will be reviewed to identify further relevant literature. All citations will be managed using Zotero Reference Manager, with duplicate records systematically identified and removed to ensure the integrity of the dataset.

### Study selection process

The study selection will follow a rigorous, three-stage procedure. All identified records will be imported into Covidence systematic review software for screening and data management [33]. Initially, all retrieved records will be consolidated and duplicates removed, alongside any citations lacking abstracts. In the second stage, two independent reviewers will screen titles and abstracts against predefined inclusion and exclusion criteria, classifying each record as “include,” “exclude,” or “uncertain.” This stage aims to maximise reliability and reduce bias, in line with best practices for systematic and scoping reviews.

Following this, in the third stage, full-text articles of all potentially eligible studies will be obtained. Two reviewers will independently assess these articles using the same coding schema. If required, study authors will be contacted to clarify or obtain missing information. Any discrepancies arising at either the title/abstract or full-text review stage will first be addressed through discussion and consensus; unresolved cases will be adjudicated by a third reviewer. We will meticulously record the reasons for excluding full-text articles and report frequencies of these exclusions in the final scoping review.

To promote consistency across reviewers, a calibration exercise will be conducted before screening begins. Regular team meetings will be held throughout the screening process to discuss ambiguous cases and maintain alignment on inclusion or exclusion decisions.

All selection and screening activities will be managed using Microsoft Excel and the Covidence software platform, enabling structured workflow, conflict tracking, and automatic generation of a PRISMA-ScR flow diagram where appropriate [28]. The methodology is designed according to the PRISMA-ScR reporting guidelines, and details can be found in S1 File.

### Data extraction

A standardised data extraction form will be developed and pilot-tested on a sample of included studies before full data extraction begins (Table 2). The data extraction form will capture study characteristics including publication details, study design, setting, participant demographics, intervention details, theoretical framework, delivery format, duration and intensity, comparison conditions, outcomes measured, key findings, and reported implementation factors. Two reviewers will independently extract data from all included studies, with discrepancies resolved through discussion.

**Table 2:** Data extraction table.

Categories	Variable
Domain 1: Study Characteristics	Author(s) Year of publication Location of study (country) Design Aims/purpose Setting and context Population
Domain 2: Population Characteristics	Sample size Groups Age range and demographic characteristics Mean/median age Sex/gender Race/ethnicity Socio-economic status Migration status and background Duration of displacement Phases of displacement Trauma exposure and mental health needs
Domain 3: Intervention Characteristics	Type and description of intervention Ecological level of intervention Theoretical framework/approach Duration, frequency, and intensity Delivery method and setting Intervention providers and training Cultural adaptations
Domain 4: Outcome measures	Primary and secondary outcomes Outcome domains Measurement instruments used Results and effectiveness data Follow-up periods
Domain 5: Implementation Factors	Facilitators and barriers Acceptability and feasibility Cost considerations Sustainability factors

Data extraction will include a dedicated component on theoretical frameworks. Reviewers will record: (a) whether a theoretical framework is explicitly named; (b) the specific framework(s) identified; (c) the extent to which the framework is operationalised, classified as fully operationalised, partially operationalised, or named only; and (d) whether the study addresses theoretical rationale for adapting the intervention to forced migration or cultural contexts. During thematic analysis, patterns across frameworks will be examined to identify the most commonly used approaches, associations between frameworks and intervention types or settings, and gaps in theoretical grounding. Studies lacking an explicit framework will be documented as atheoretical and reported, given that this has been identified as a notable gap in prior literature [18,20]. This information will be captured in the theoretical framework category of the data extraction table (Table 2).

### Quality assessment

As recommended for scoping reviews, formal quality assessment tools will not be applied. This is consistent with JBI methodological guidance, which specifies that critical appraisal is not a mandatory component of the scoping review process, given its purpose of mapping evidence rather than synthesising its quality [27]. However, methodological characteristics and limitations of included studies will be documented during data charting. This will provide context for interpreting findings and identifying areas where higher-quality research is needed.

### Data analysis and presentation

Following the PRISMA-ScR guidelines, the number of studies at each stage of the review will be recorded. The studies included will undergo thorough data analysis and tabulation. Data will be analysed using a narrative synthesis and thematic analysis approach. Narrative synthesis will be used to summarise and map the descriptive characteristics of included studies and interventions. Quantitative data will be summarised using descriptive statistics where appropriate, while qualitative findings will be synthesised thematically. This approach will be used specifically to analyse: (a) reported facilitators and barriers to intervention implementation across different contexts and settings; (b) patterns in how interventions have been adapted or modified for different cultural, linguistic, or displacement contexts; (c) reported challenges and strategies in engaging adolescent populations in forced migration settings; and (d) gaps and inconsistencies in theoretical frameworks underpinning interventions. The authors will work together to analyse the quotations thematically, using procedures described by Braun and Clarke [34]. Analysis will be supported by qualitative data analysis software NVivo. We will complete this process reflexively by constantly asking ourselves, and each other, how our preconceptions are influencing the analytical process. We will achieve the aim of the study by presenting these findings, supported by illustrative quotations. The review will map the characteristics of available evidence, including study designs, populations, interventions, and outcomes, to identify patterns and gaps in the literature.

### Ethical considerations

As the review is based on previously published literature, formal ethical approval is not required. However, the review will adhere to established ethical principles, including respect for the dignity of participants in the original studies, acknowledgement of researcher positionality, and consideration of potential harms. Care will be taken in the interpretation and application of the findings.

### Protocol registration

This protocol has been registered with the Open Science Framework (OSF) with DOI: 10.17605/OSF.IO/ADF92. This registration will ensure transparency and reduce duplication of effort.

### Dissemination plan

Results will be disseminated through publication in a peer-reviewed journal, presentations at relevant academic and professional conferences, and sharing with key stakeholders, including humanitarian organisations and policy makers. The review findings will also inform the development of future systematic reviews and meta-analyses examining specific intervention types or outcomes in greater detail.

The completed review will contribute to the evidence base supporting effective psychosocial care for one of the world’s most vulnerable populations, ultimately supporting improved mental health and wellbeing outcomes for adolescents affected by forced migration.

## Conclusion

This scoping review will provide a comprehensive mapping of psychosocial interventions specifically designed for adolescents in forced migration, thereby addressing a critical research gap. The timing is particularly relevant given recent global displacement events and increased attention to refugee mental health, combined with growing recognition of climate change’s contribution to new migration patterns [35]. A significant strength is the protocol’s attention to implementation factors and barriers, which are often overlooked in traditional efficacy-focused reviews. Understanding how interventions are delivered, what facilitates or impedes implementation, and how they adapt to different contexts is crucial for translating research into practice. The review’s emphasis on theoretical frameworks addresses another literature gap, as many interventions are developed pragmatically without explicit grounding in developmental or trauma theory. By systematically examining theoretical foundations, the review may identify patterns that inform future intervention development and explain why certain approaches succeed in certain contexts. Therefore, this review will provide valuable guidance for the implementation of evidence-based interventions in resource-constrained or politically sensitive contexts.

## Supporting information

S1 File Preferred Reporting Items for Systematic reviews and Meta-Analyses extension for Scoping Reviews (PRISMA-ScR) Checklist. (DOCX)

## Abbreviations

IDPs: Internally Displaced Persons
SGBV: Sexual and Gender-Based Violence
PTSD: Post-Traumatic Stress Disorder
CBT: Cognitive-Behavioural Therapy
TIC: Trauma-Informed Care
NET: Narrative Exposure Therapy
FBIs: Family-Based Interventions
CBPs: Community-Based Psychosocial Support
SBMNP: School-Based Mental Health Pro
IASC: Interagency Standing Committee (IASC) Guidelines on Mental Health and Psychosocial Support in Emergency Settings
Prospero: International Prospective Register of Systematic Reviews
CINAHL: Cumulative Index of Nursing and Allied Health Literature
PCC: Participants-Concept–Context
JBI: Joanna Briggs Institute
PRISMA-SCR: Preferred Reporting Items for Systematic reviews and Meta-Analyses extension for Scoping Reviews
MESH: Medical Subject Headings
MEDLINE: Medical Literature Analysis and Retrieval System Online
OVID: Psychological Information Database
Embase: Excerpta Medica database
UNHCR: United Nations High Commission for Refugees
WHO: World Health Organization
OSF: Open Science Framework

## References

[1] Alcock M. Refugee trauma — the assault on meaning. Psychodyn Pract. 2003;9(3):291–306. doi: 10.1080/1353333031000139255

[2] Refugee Data Finder - Key Indicators [Internet]. [cited 2025 Sep 4]. Available from: https://www.unhcr.org/refugee-statistics

[3] Adolescent health - SEARO [Internet]. [cited 2025 Aug 20]. Available from: https://www.who.int/southeastasia/health-topics/adolescent-health

[4] Pfeifer JH, Berkman ET. The development of self and identity in adolescence: neural evidence and implications for a value-based choice perspective on motivated behavior. Child Dev Perspect. 2018;12(3):158–64. doi: 10.1111/cdep.12279 31363361 PMC6667174

[5] Rogers-Sirin SRSL. The Educational and Mental Health Needs of Syrian Refugee Children. 2015 [cited 2025 Jul 31]. Available from: https://www.migrationpolicy.org/research/educational-and-mental-health-needs-syrian-refugee-children

[6] Montgomery E. Trauma and resilience in young refugees: a 9-year follow-up study. Dev Psychopathol. 2010;22(2):477–89. doi: 10.1017/S0954579410000180 20423554

[7] UNHCR [Internet]. Global Trends. [cited 2025 Aug 1]. Available from: https://www.unhcr.org/global-trends

[8] Available from: https://genocidescholars.org/wp-content/uploads/2025/08/IAGS-Resolution-on-Gaza-FINAL.pdf

[9] Murthy RS, Lakshminarayana R. Mental health consequences of war: a brief review of research findings. World Psychiatry. 2006;5(1):25–30. 16757987 PMC1472271

[10] Mental Health in Displaced Child and Youth Populations: A developmental and family systems lens | Innocenti Global Office of Research and Foresight [Internet]. 2023 [cited 2025 Aug 19]. Available from: https://www.unicef.org/innocenti/reports/mental-health-displaced-child-and-youth-populations

[11] Betancourt TS, Newnham EA, Layne CM, Kim S, Steinberg AM, Ellis H, et al. Trauma history and psychopathology in war-affected refugee children referred for trauma-related mental health services in the United States. J Trauma Stress. 2012;25(6):682–90. doi: 10.1002/jts.21749 23225034

[12] Bronstein I, Montgomery P. Psychological distress in refugee children: a systematic review. Clin Child Fam Psychol Rev. 2011;14(1):44–56. doi: 10.1007/s10567-010-0081-0 21181268

[13] Patton GC, Sawyer SM, Santelli JS, Ross DA, Afifi R, Allen NB. Our future: a Lancet commission on adolescent health and wellbeing. Lancet. 2016;387(10036):2423–78. doi: 10.1016/S0140-6736(16)00579-1 27174304 PMC5832967

[14] Fazel M, Reed RV, Panter-Brick C, Stein A. Mental health of displaced and refugee children resettled in high-income countries: risk and protective factors. Lancet. 2012;379(9812):266–82. doi: 10.1016/S0140-6736(11)60051-2 21835459

[15] Refugee and migrant mental health [Internet]. [cited 2025 Aug 19]. Available from: https://www.who.int/news-room/fact-sheets/detail/refugee-and-migrant-mental-health

[16] Moutsou I, Georgaca E, Varaklis T. Psychotherapeutic and psychosocial interventions with unaccompanied minors: a scoping review. Healthcare. 2023;11(6):918. doi: 10.3390/healthcare11060918 36981575 PMC10048295

[17] IASC Guidelines on Mental Health and Psychosocial Support in Emergency Settings, | IASC [Internet]. 2007 [cited 2025 Jul 31]. Available from: https://interagencystandingcommittee.org/iasc-task-force-mental-health-and-psychosocial-support-emergency-settings/iasc-guidelines-mental-health-and-psychosocial-support-emergency-settings-2007

[18] Frounfelker RL, Miconi D, Farrar J, Brooks M, Rousseau C, Betancourt TS. Mental health of refugee children and youth: epidemiology, interventions and future directions. Ann Rev Public Health. 2020;41:159–76. doi: 10.1146/annurev-publhealth-040119-094230 31910713 PMC9307067

[19] Purgato M, Gross AL, Betancourt T, Bolton P, Bonetto C, Gastaldon C, et al. Focused psychosocial interventions for children in low-resource humanitarian settings: a systematic review and individual participant data meta-analysis. Lancet Glob Health. 2018;6(4):e390–400. doi: 10.1016/S2214-109X(18)30046-9 29530422

[20] Jordans MJD, Pigott H, Tol WA. Interventions for children affected by armed conflict: a systematic review of mental health and psychosocial support in low- and middle-income countries. Curr Psychiatry Rep. 2016;18(1):9. doi: 10.1007/s11920-015-0648-z 26769198 PMC4713453

[21] Howard K, Moore D, Dimitrellou E, Janik Blaskova L, Howard J. School-based mental health support for migrant children and young people: a scoping review. J Sch Psychol. 2024;107:101393. doi: 10.1016/j.jsp.2024.101393 39645329

[22] Turrini G, Purgato M, Acarturk C, Anttila M, Au T, Ballette F, et al. Efficacy and acceptability of psychosocial interventions in asylum seekers and refugees: systematic review and meta-analysis. Epidemiol Psychiatr Sci. 2019;28(4):376–88. doi: 10.1017/S2045796019000027 30739625 PMC6669989

[23] Hettich N, Seidel FA, Stuhrmann LY. Psychosocial interventions for newly arrived adolescent refugees: a systematic review. Adolescent Res Rev. 2020;5(2):99–114. doi: 10.1007/s40894-020-00134-1

[24] Tyrer RA, Fazel M. School and community-based interventions for refugee and asylum seeking children: a systematic review. PLoS One. 2014;9(2):e89359. doi: 10.1371/journal.pone.0089359 24586715 PMC3933416

[25] Bennouna C, Khauli N, Basir M, Allaf C, Wessells M, Stark L. School-based programs for Supporting the mental health and psychosocial wellbeing of adolescent forced migrants in high-income countries: a scoping review. Soc Sci Med. 2019;239:112558. doi: 10.1016/j.socscimed.2019.112558 31539785

[26] Arksey H, O’Malley L. Scoping studies: towards a methodological framework. Int J Soc Res Methodol. 2005;8(1):19–32. doi: 10.1080/1364557032000119616

[27] Peters MDJ, Marnie C, Tricco AC, Pollock D, Munn Z, Alexander L, et al. Updated methodological guidance for the conduct of scoping reviews. JBI Evid Synth. 2020;18(10):2119–26. doi: 10.11124/JBIES-20-00167 33038124

[28] Tricco AC, Lillie E, Zarin W, O’Brien KK, Colquhoun H, Levac D, et al. PRISMA Extension for Scoping Reviews (PRISMA-ScR): Checklist and Explanation. Ann Intern Med. 2018;169(7):467–73. doi: 10.7326/M18-0850 30178033

[29] Adolescent health [Internet]. [cited 2025 Jul 22]. Available from: https://www.who.int/health-topics/adolescent-health

[30] Barbui C, Purgato M, Abdulmalik J, Acarturk C, Eaton J, Gastaldon C, et al. Efficacy of psychosocial interventions for mental health outcomes in low-income and middle-income countries: an umbrella review. Lancet Psychiatry. 2020;7(2):162–72. doi: 10.1016/S2215-0366(19)30511-5 31948935

[31] Tol WA, Purgato M, Bass JK, Galappatti A, Eaton W. Mental health and psychosocial support in humanitarian settings: a public mental health perspective. Epidemiol Psychiatr Sci. 2015;24(6):484–94. doi: 10.1017/S2045796015000827 26399635 PMC8367376

[32] Booth A. Unpacking your literature search toolbox: on search styles and tactics. Health Info Libr J. 2008;25(4):313–7. doi: 10.1111/j.1471-1842.2008.00825.x 19076679

[33] Covidence - Better systematic review management [Internet]. [cited 2025 Jul 22]. Available from: https://www.covidence.org/

[34] Braun V, Clarke V. Using thematic analysis in psychology. Qual Res Psychol. 2006;3(2):77–101. doi: 10.1191/1478088706qp063oa

[35] Della Rocca B, Bello R, Carbone M, Pezzella P, Toni C, Sampogna G, et al. Promoting mental health and preventing mental health problems in child and adolescent refugees and asylum seekers: a systematic review on psychosocial interventions. Int J Soc Psychiatry. 2024;70(4):653–66. doi: 10.1177/00207640231214964 38069651

